# Potential hemo-biological identification markers to the left displaced abomasum in dairy cows

**DOI:** 10.1186/s12917-020-02676-x

**Published:** 2020-12-02

**Authors:** Yuxiang Song, Juan J Loor, Chenchen Zhao, Dan Huang, Xiliang Du, Xiaobing Li, Zhe Wang, Guowen Liu, Xinwei Li

**Affiliations:** 1 Key Laboratory of Zoonosis Research, Ministry of Education, College of Veterinary Medicine, Jilin University, 5333 Xi’an Road, Jilin 130062 Changchun, China; 2 Mammalian NutriPhysioGenomics, Department of Animal Sciences, Division of Nutritional Sciences, University of Illinois, 61801 Urbana, USA

**Keywords:** Left displaced abomasum, Hematological biomarkers, Dairy cow, Disease identification, Negative energy balance, ROC curve

## Abstract

**Background:**

Left displaced abomasum (LDA) occurs at high frequency in the early postpartum period and can affect production performance of dairy cows. Clinical diagnosis of LDA is usually done by abdominal auscultation and percussion. The purpose of this study was to explore the potential applicability of blood biomarkers for early warning and diagnosis of LDA in dairy cows.

**Results:**

Twenty early postpartum healthy cows and thirty early postpartum LDA cows of similar parity were used. A receiver operating characteristic curve (ROC) method was used to analyze the sensitivity of hematological biomarkers to LDA including energy balance metabolic biomarkers, liver/kidney function biomarkers, and minerals. A cut-off point was defined for each of the selected hematological biomarkers deemed sensitive markers of LDA. Compared with healthy cows, body condition score (BCS), dry matter intake (DMI) and milk production were lower in LDA cows. Among energy metabolism markers, serum non-esterified fatty acid (NEFA), β-hydroxybutyric acid (BHBA), insulin (INS), and revised quantitative insulin sensitivity check index (RQUICKI) levels were lower while serum glucagon (GC) was greater in LDA cows. Among the liver/kidney function biomarkers, activities of serum alanine aminotransferase (ALT), aspartate aminotransferase (AST), γ-glutamyl transpeptidase (GGT), lactate dehydrogenase (LDH), the ratio of AST/ALT and levels of total bilirubin (TBIL), direct bilirubin (DBIL), indirect bilirubin (IBIL), albumin (ALB), blood urea nitrogen (BUN), creatinine, and total protein (TP) were greater in LDA cows. Among minerals analyzed, serum Cl, Ca, and K were lower in LDA cows. After ROC analysis, it was determined that serum Ca, INS, RQUICKI, ALT, GGT, and creatinine are potential indicators for early warning and diagnosis of LDA for early postpartum dairy cows.

**Conclusions:**

Dairy cows with LDA were under severe negative energy balance (NEB), had signs of liver damage and potentially lower insulin sensitivity. A combination of multi-hematological biomarkers including Ca, INS, RQUICKI, ALT, GGT and creatinine has the potential to help identify cows at risk of LDA in the early postpartum period.

## Background

Displacement of the abomasum in dairy cattle is a multifactorial disease and has become a major problem in the modern dairy industry [1]. Although the mechanisms behind abomasum displacement are not completely known, there is evidence that stress conditions, nutrition, and metabolic disturbances all contribute to the disorder [2, 3]. Other contributing factors are those causing weakened abomasal motility including metabolic disorders (hypocalcemia and ketosis), concurrent diseases (mastitis, metritis, retained placenta, and subclinical milk fever), genetic predisposition, and lack of exercise [4, 5]. Left displaced abomasum (LDA) is seen much more frequently than right abomasal displacement [1]. In developing countries such as China where there is a growing number of large dairy farms, economic loss associated with LDA are becoming more tangible. Thus, it is necessary to improve the early warning and diagnostic ability for LDA especially in large dairy farms.

Simultaneous auscultation and percussion or ballottement on the left mid-flank area of the abdomen are traditional diagnostic methods. The LDA can be detected clinically if gas is present in the abomasum with a tympanic, resonant, high-pitched ‘pinging’ sound [6]. Although a diagnosis usually can be made directly by the specific ‘pinging’, some intensive methods such as rectal examination, blowing air into the rumen through a stomach tube or abomasocentesis, are required to differentiate rumen collapse syndrome, rumen tympany and peritonitis pneumoperitoneum from LDA [7]. In addition, some large-scale modern dairy farms are equipped with B-mode ultrasonic apparatus for large animals, helping the diagnosis of LDA [6]. The treatment of LDA can be either conservative (casting and rolling the cow) or surgical. Both conservative and surgical therapies help manipulate the abomasum returns to its normal position. Compared to conservative therapy, surgical therapy reduces the risk of recurrence and allows assessment of the condition of the abomasum. However, more attention should be paid to the secondary infection and the reduction of production performance caused by surgery [2, 6].

Previous studies reported that hematological biomarkers are useful diagnostic indicators of abdominal disorders because these biomarkers could reflect the metabolism, stress, injury and inflammatory conditions in dairy cows [8]. Importantly, in humans, hematological biomarkers analysis has been widely used in the early prediction and warning of metabolic diseases including insulin resistance, body mass index (BMI), fatty liver, and obesity [9]. The diagnostic efficacy of these sensitive hematological biomarkers for metabolic diseases are often compared using a receiver operating characteristic curve (ROC) analysis. The ROC analysis is a valuable statistical tool, which evaluates the sensitivity and the specificity of biomarkers to be used in making a diagnostic decision. For instance, Abruzzo et al. [10] reported that a ROC curve should become the gold standard for evaluation of sensitivity and specificity of biomarkers to support disease diagnosis, risk assessment, as well as therapeutic interventions [10].

Although previous work has used biomarkers associate with the acute-phase response, oxidative stress and hepatic function for LDA diagnosis [4] the use of energy metabolism, renal function biomarkers, and minerals have not been studied in the context of LDA. More importantly, previous studies have not defined cut-off points for those indicators deemed sensitive, which would be helpful for the early warning and identification of cows predisposed to LDA. Therefore, this study aimed to investigate the levels or activity of hematological biomarkers reflecting energy metabolism, hepatic and renal function and mineral element balance to identify sensitive indicators for LDA identification in dairy cows.

## Results

### General characteristic of cows

Cows with LDA displayed significantly lower body condition score (BCS) and DMI (~ 65% decrease) compared with healthy cows. Importantly, milk production of LDA cows was also lower compared with healthy cows (Table 1).

**Table 1:** The general characteristic of the LDA and control dairy cows

Group	BCS	Milk production (kg/day)	DMI (kg/day)
Control	3.00±0.06	31.65±0.46	15.92±0.15
LDA	2.5±0.05*	22.95±0.09**	5.01±0.05**

The general characteristic of LDA and control dairy cows were shown in the table, including parity, BCS, milk production and DMI. Healthy Holstein cows, *n* = 20; LDA Holstein cows, *n* = 30. The results are expressed as the mean ± SEM. *P < 0.05, **P < 0.01, compared with respective values in control (healthy) group

*BCS* body condition score, *DMI *dry matter intake

### Analysis of blood energy metabolism biomarkers

Compared with healthy cows, no difference was found for serum GLU, TC and TG levels (Table 2). However, LDA cows had ~ 3 times the serum NEFA level and ~ 2.5 times the serum BHBA level (*P* < 0.01) compared with healthy cows, suggesting they were in more pronounced negative energy balance (NEB). In addition, although the serum GLU was not affected by LDA, serum INS and RQUICKI were lower while serum GC level greater in LDA cows. Thus, NEFA, BHBA, INS, GC, and RQUICKI were selected as potential biomarkers for analysis by the ROC method.

**Table 2:** Blood energy metabolism-related biomarkers in dairy cows with LDA or healthy cows

Detected biomarkers	Control		LDA		*p*
	Mean±SEM	95% CI	Mean±SEM	95% CI	
GLU (mmol/L)	4.31±0.13	3.76-4.81	4.84±0.42	4.44-5.33	0.487
NEFA (mmol/L)	0.36±0.04	0.31-0.41	0.99±0.06**	0.92-1.22	0.000
BHBA (mmol/L)	0.45±0.03	0.37-0.55	1.17±0.24**	1.03-1.87	0.003
INS (mU/L)	12.68±0.28	7.99-13.26	9.05±0.21**	4.31-6.34	0.000
GC (ng/L)	48.26±1.63	42.67-51.36	55.56±0.90*	52.55-58.79	0.040
RQUICKI	0.79±0.04	0.62-0.85	0.60±0.02**	0.55-0.67	0.002
TC (mmol/L)	2.42±0.24	1.91-2.92	1.96±0.16	1.63-2.27	0.120
TG (mmol/L)	0.16±0.01	0.14-0.18	0.15±0.01	0.12-0.16	0.263

The serum energy metabolism-related biomarkers of dairy cows with LDA or healthy cows (Control) were shown in the table. Healthy Holstein cows, *n* = 20; LDA Holstein cows, *n* = 30. The results are expressed as the mean ± SEM. *P < 0.05, **P < 0.01, compared with respective values in control group

*GLU* Glucose, *NEFA* Non-esterified fatty acid, *BHBA* β-hydroxybutyric acid, *INS* Insulin, *GC* Glycagon, *RQUICKI* Revised quantitative insulin sensitivity check index, *TC* Total cholesterol, *TG* Triglyceride

### Analysis of liver/kidney function-related biomarkers

Activities of ALT, AST, GGT, LDH, ratio of AST/ALT and levels of TBIL, DBIL and IBIL were greater in LDA compared with healthy cows (Table 3). However, lower ALB and TP were detected in LDA cows. Concentrations of BUN and creatinine were greater in LDA cows. Thus, ALT, AST, GGT, LDH, TBIL, BBIL, IBIL, TP, ALB, BUN, and creatinine were selected as potential biomarkers for analysis by the ROC method.

**Table 3:** Liver/kidney function related biomarkers in dairy cows with LDA or healthy cows

Detected biomarkers	Control		LDA		p
	Mean±SEM	95% CI	Mean±SEM	95% CI	
ALT (U/L)	19.20±1.42	16.82-21.57	23.20±1.16**	20.82-25.57	0.000
AST (U/L)	86.65±3.18	80.00-93.29	122.87±6.65**	109.27-136.46	0.000
AST/ALT	4.48±0.26	3.92-5.03	6.70±0.35**	5.98-7.42	0.000
GGT (U/L)	23.20±2.56	20.33-25.87	32.07±2.22**	28.52-35.61	0.015
LDH (U/L)	850.37±63.5	744.05-956.68	950.37±51.92*	844.05-1056.68	0.015
TBIL (μmol/L)	4.07±0.5	3.02-5.11	6.30±0.52**	5.23-7.37	0.002
DBIL (μmol/L)	1.54±0.19	1.13-1.95	2.48±0.22**	2.02-2.94	0.001
IBIL (μmol/L)	2.53±0.31	1.87-3.18	3.82±0.34*	3.13-4.51	0.010
TP (g/L)	76.05±1.35	73.23-78.87	68.74±1.81**	65.04-72.43	0.040
ALB (g/L)	33.78±0.66	32.38-35.16	27.35±0.67*	25.98-29.71	0.005
GLO (g/L)	42.27±1.41	39.31-45.22	39.39±1.55	36.21-42.56	0.201
BUN (mmol/L)	4.61±0.3	3.99-5.23	6.20±0.52*	5.14-7.26	0.040
Creatinine (mmol/L)	63.00±2.12	58.55-67.45	79.03±3.4**	72.07-85.99	0.001

The Liver/kidney function related biomarkers of dairy cows with LDA or healthy cows (Control) were shown in the table. Healthy Holstein cows, *n* = 20; LDA Holstein cows, *n* = 30. The results are expressed as the mean ± SEM. *P < 0.05, **P < 0.01, compared with respective values in control (healthy) group

*ALT* Alanine aminotransferase, *AST* Aspartate aminotransferase, *GGT* γ glutamyl transpeptidase, *LDH* Lactate dehydrogenase, *TBIL* Total bilirubin, *DBIL* Direct bilirubin, *IBIL* Indirect bilirubin, *TP* Total protein, *ALB* Albumin, *GLO* Globulin, *BUN* Blood urea nitrogen

### Analysis of blood minerals

No significant difference was detected in serum Na, P, Mg, Cu and Zn levels due to LDA (Table 4). However, compared with healthy cows, serum Cl, Ca and K levels were lower due to LDA. Thus, Cl, Ca and K were selected as potential biomarkers for analysis by the ROC method.

**Table 4:** Minerals in dairy cows with LDA or healthy cows

Detected biomarkers	Control		LDA		p
	Mean±SEM	95% CI	Mean±SEM	95% CI	
Na (mmol/L)	139.07 ±1.16	136.63-141.50	134.96 ±1.24	132.43-137.49	0.026
Cl (mmol/L)	103.15 ±0.89	101.77-105.51	95.67 ±0.46**	93.24-98.10	0.000
Ca (mmol/L)	2.41 ±0.03	2.36-2.47	2.07 ±0.04**	1.98-2.16	0.000
P (mmol/L)	2.01 ±0.06	1.87-2.14	1.81 ±0.12	1.57-2.05	0.203
Mg (mmol/L)	1.09 ±0.02	1.04-1.14	1.03 ±0.03	0.97-1.08	0.075
K (mmol/L)	5.14 ±0.17	4.437-5.650	3.76 ±0.31**	3.35-4.16	0.000
Cu (μmol/L)	20.57 ±0.33	19.88-21.26	19.47 ±0.91	17.61-21.24	0.344
Zn (μmol/L)	6.71 ±0.42	6.21-7.10	6.48 ±0.2	6.01-6.74	0.450

The minerals of dairy cows with LDA or healthy cows (Control) were shown in the table. Healthy Holstein cows, *n* = 20; LDA Holstein cows, *n* = 30. The results are expressed as the mean ± SEM. *P < 0.05, **P < 0.01, compared with respective values in control (healthy) group

### Efficacy of biomarkers for LDA identification

**Fig. 1:**
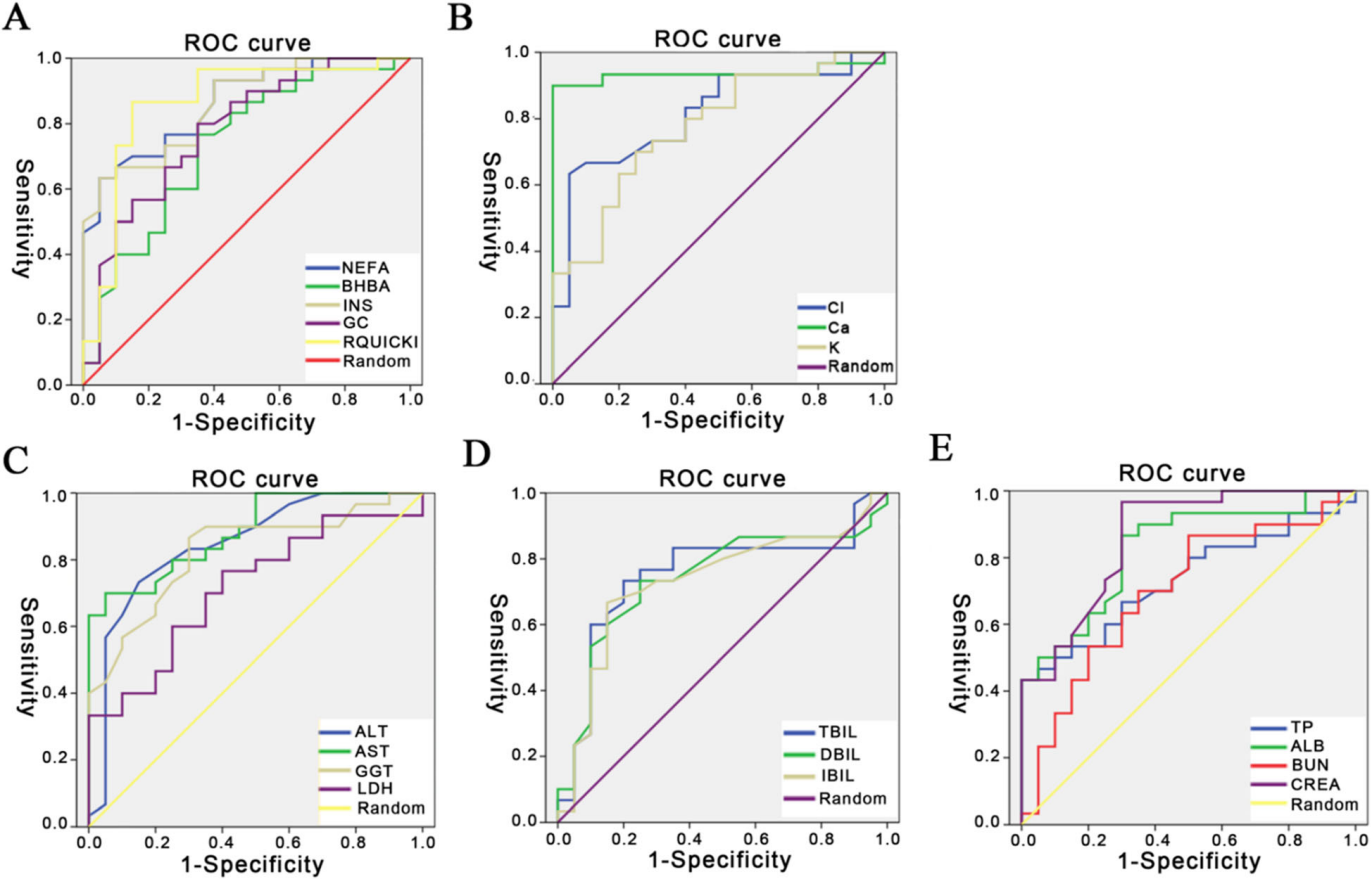
ROC curves of potential biomarkers for LDA identification. ROC curves were constructed to warn LDA in dairy cows. **a** ROC of energy metabolism-related biomarkers; **b** ROC of minerals; **c-d-e**) ROC of liver/kidney function-related biomarkers. To clearly show the ROC curve of each parameter, the data were shown in separated images with different colors. The random line in each image stands for a reference line. ROC (receiver operating characteristic curve); Creatinine (CREA)

The ROC curves are shown separately in Fig. 1 and the corresponding AUC, sensitivity, specificity, and cut-off points are listed in Table 5. Among these biomarkers, AUC for BHBA (0.75), GC (0.73), LDH (0.71), TBIL (0.75), IBIL (0.73), TP (0.73) and BUN (0.7) were lower (below 0.8) while AUC for NEFA (0.84), INS (0.89), RQUICKI (0.89), ALT (0.89), AST (0.84), GGT (0.88), ALB (0.82), and creatinine (0.86) were greater (over 0.8) (Fig. 1a and c). Estimated cut-off points were NEFA-0.68 mM, INS-10.4 mU/L, RQUICKI-0.68, ALT-19.5 U/L, AST-106 U/L, GGT-24.5 U/L, ALB-33 µM, and creatinine-74 mM (Table 5). These data suggested that energy metabolism-related biomarkers including NEFA, INS, and RQUICKI, as well as liver/kidney function-related biomarkers including ALT, AST, GGT, ALB, and creatinine are effective for LDA identification. The ROC curves for minerals are shown in Fig. 1b. The AUC for Ca was 0.94 and for Cl 0.81 (Table 5) suggesting that serum Ca and Cl also were effective biomarkers for LDA identification with a cut-off point of 2.25 mM and 98.01 mM, respectively (Table 5).

**Table 5:** AUC, sensitivity and specificity of potential biomarkers for LDA identification

	Energy metabolism biomarkers					Liver/kidney function biomarkers											Minerals		
	NEFA	BHBA	INS	GC	RQUICKI	ALT	AST	GGT	LDH	TBIL	DBIL	IBIL	TP	ALB	BUN	Creatinine	Cl	Ca	K
**AUC**	0.84	0.75	0.89	0.73	0.89	0.89	0.84	0.88	0.71	0.75	0.74	0.73	0.73	0.82	0.7	0.86	0.81	0.94	0.78
**Se**	0.77	0.8	0.87	0.73	0.83	0.87	0.7	0.87	0.77	0.73	0.73	0.67	0.43	0.87	0.87	0.97	0.63	0.90	0.70
**Sp**	0.9	0.65	0.85	0.65	0.85	0.85	0.95	0.75	0.6	0.8	0.75	0.85	0.95	0.7	0.5	0.7	0.95	0.95	0.75
**Cp**	0.68 mM	0.75 mM	10.4 mU/L	58.6 ng/L	0.68	19.5 U/L	106 U/L	24.5 U/L	812 U/L	4.85 μM	1.65 μM	2.95 μM	67.6 μM	33.0 μM	5.34 mM	74 mM	98 mM	2.25 mM	4.05 mM

Each evaluated variable of the potential biomarkers, including area under the curves (AUCs), sensitivity, specificity, or cut-point, was calculated and shown in the table. Youden Index (YI)was calculated as YI = Sensitivity- (1-specificity). A cut-point for each parameter was chosen when a maximum YI was detected

*Se* Sensitivity, *Sp* Specificity, *Cp* Cut point, *NEFA* Non-esterified fatty acid, *BHBA* β-hydroxybutyric acid, *INS* Insulin, *GC* Glycagon, *RQUICKI* Revised quantitative insulin sensitivity check index, *ALT* Alanine aminotransferase, *AST* Aspartate aminotransferase, *GGT* γ glutamyl transpeptidase, *LDH* Lactate dehydrogenase, *TBIL* Total bilirubin, *DBIL* Direct bilirubin, *IBIL* Indirect bilirubin, *TP* Total protein, *ALB* Albumin, *BUN* Blood urea nitrogen

## Discussion

Displaced abomasum is more frequent in high producing cows with more than 80% of cases occuring within 3–4 weeks postpartum [12], a period that coincides with negative energy balance (NEB) resulting from increased energy needs and reduced dry matter intake. These have been suggested to be a risk factor for LDA [11]. The consensus of opinion has it that increased serum NEFA and BHBA levels, as blood characteristics of NEB cows, are associated with an increased risk of LDA [12–14]. Consistent with the previous studies [8], we confirmed the strong increase in serum NEFA and BHBA levels in LDA cows. Findings regarding GLU and INS levels in the published literature have been inconsistent in LDA cows. For instance, it has been reported that cows with displaced abomasum had elevated serum GLU and INS levels [5, 14]. The reason why abomasal displacement in these studies led to hyperglycemia is still unknown. Van Winden et al. [3] found decreased serum INS and GLU levels in cows developing abomasum displacement after 10 days from calving. The state of NEB is characterized by low serum GLU and INS levels along with elevated serum NEFA and BHBA levels [15]. Possible explanations for these opposite results include the uncertain duration of LDA onset at the detection and the additional environmental stress during transportation [16].

In the present study, the environmental interference was reduced, and serum GLU level in healthy and LDA cows agree with [16]. The lower RQUICKI in LDA cows in our study suggested they experienced a more pronounced degree of insulin resistance. The RQUICKI, an index based on serum NEFA, GLU, and INS, was used to determine insulin sensitivity in both humans [17, 18] and cows [16, 19, 20]. Thus, the RQUICKI can be a suitable candidate for LDA identification.

Metabolism of circulating NEFA occurs mainly in the liver, where they can be completely oxidized for energy, exported as lipoproteins, or partially oxidized into ketone bodies such as BHBA [21, 22]. Enhanced lipid mobilization will result in an overload of NEFA in the liver and further accumulation of ketone bodies, eventually leading to subclinical or clinical ketosis [23]. High serum NEFA or ketone bodies induce hepatocyte damage [24–26]. The activities of ALT, AST, GGT and LDH reflect the integrity of hepatocytes and are often regarded as sensitive indicators of liver injury [27, 28]. Thus, the greater activities of all these enzymes in LDA cows suggested the existence of hepatocytes damage, which is consistent with previous studies [4, 8, 16].

Bilirubin is another sensitive parameter for evaluating functional capacity of the liver. The concentration of serum bilirubin is closely related to liver injury or bile duct abnormality. For example, dilation of the gallbladder duct is often detected in cows with LDA [14, 29]. Thus, the greater serum TBIL, DBIL and IBIL in LDA cows was consistent with previous studies, underscoring the existence of liver damage or bile duct obstruction in cows with LDA [8]. One explanation is that hyperbilirubinemia in LDA cows may be caused by biliary traction due to the change of duodenal position [14]. The level of serum TP can reflect the state of water/salt metabolism and the reserve capacity of the liver. Decreased reserve capacity of the liver often follows liver damage, which can be manifested by a decrease of serum TP and ALB levels. Thus, the lower TP and ALB levels in cows with LDA in the present study were consistent with the literature [8].

BUN and creatinine are the final product of protein metabolism and they are often regarded as indicators of renal function. The greater BUN and creatinine in LDA cows was consistent with the findings of de Cardoso et al. [8]. The nephrogenic increase of both happens only when the glomerular filtration rate falls below 50%. We are unaware of previous studies reporting impaired renal function in cows with LDA. Alternatively, the increase of BUN and creatinine can also result from increased protein decomposition or dehydration, which is more likely in the present study as judged by haemoconcentration and dehydration in cows with LDA [30]. Overall the present and previous data indicate that liver- or kidney-related biomarkers are tightly associated with LDA in dairy cows. Thus, they should be under consideration as serum indicators for early warning.

High amounts of K are lost through milk production (1.4 g K per liter of milk), which is a reason why high-yielding dairy cows usually have low serum K concentrations [31, 32]. Alternatively, K also participates in the metabolism and synthesis of glycogen and protein [33], and these processes are enhanced in LDA dairy cows undergoing NEB [34]. It has been shown that K and Cl are not transported from the abomasum into the duodenum, but flow back into the forestomach in cows with LDA [5]. In addition to the above factors, reduced food intake in LDA cows, resulting in decreased K intake, should also be taken into consideration.

A strong positive correlation between serum Cl level and K levels in dairy cows with LDA has been reported [34]. Blockage of abomasal emptying in LDA cows will result in accumulation of Cl in the rumen, which impairs the absorption of Cl and leads to alkalemia [35, 36]. The reduction of serum Cl level in LDA cows is also associated with decreased food intake. A lower serum Ca concentration in LDA cows was reported in a previous study [37]. In addition, serum Ca concentration was positively correlated with serum K concentration, both of which were negatively correlated with serum bilirubin [34]. Disequilibrium of milk-production-associated Ca consumption, dietary intake and bone Ca mobilization likely are the main causes of reductions in circulating Ca concentrations in cows with LDA. The lower serum Ca levels in cows with LDA is consistent with the study of Mokhber Dezfouli et al. [5], supporting the theory of disturbed Ca homeostasis. It is generally accepted that low serum Ca inhibits abomasal motility and promotes the occurrence of LDA in dairy cows [38]. Thus, it is meaningful to verify whether these sensitive ions can be used as indicators for LDA identification and early warning. However, the use of blood Ca or K concentration to identify LDA needs to pay special attention to differentiate from milk fever (postparturient hypercalcemia) and postparturient hypokalemia by combining other LDA blood identification indicators.

The most common diagnostic physical finding of LDA in cows is a pinging sound through simultaneous auscultation and percussion of the abdomen area marked by a line from the tuber coxae to the point of the elbow [5, 30, 39]. The analysis of hematological biomarkers is also regarded as a useful diagnostic method in abdominal disorders of dairy cows because these biomarkers could reflect the conditions of metabolism, stress, injury, and inflammation. These are of great significance in the rapid early risk warning and diagnosis of LDA, especially in large-scale dairy farms. In present study, the ROC method indicated that energy metabolism-related biomarkers including NEFA, INS, and RQUICKI; liver/kidney function-related parameters including ALT, AST, GGT, ALB, and creatinine; as well as minerals including Ca and Cl are potentially effective for LDA identification. Cut-off points also were identified. For example, serum Ca had the highest AUC (0.94) and together with RQUICKI (0.89), ALT (0.89), INS (0.89), GGT (0.88), and creatinine (0.86) can be used as preferred indicators of LDA identification for early postpartum dairy cows.

Several electronic hand-held devices have become available for easy detection of hematological biomarkers, hence, they represent excellent tools for comparing the correlation between laboratory detected data and hand-held meter measured data [40–42]. In the future, it is likely that more precise hand-held devices will be developed for specific applicability to a variety of hematological biomarkers. Our findings also provide data support for the development of these devices for LDA diagnosis and early warning.

## Conclusions

In conclusion, dairy cows with LDA were under NEB status and had disrupted Ca balance, liver damage and potentially decreased insulin sensitivity. As a supplementary method to the traditional clinical diagnosis, a combination of multi-hematological biomarkers including Ca, RQUICKI, ALT, INS, GGT and creatinine could be useful for LDA identification and early warning in early postpartum dairy cows. Further research should be performed to verify the usefulness of these indicators in actual diagnosis and early warning of LDA for early postpartum dairy cows.

## Methods

### Animals

The present study protocol was approved by the Ethics Committee on the Use and Care of Animals at Jilin University (Changchun, China) (No. 20160107). The animals received humane care according to the principles and guidelines on the ‘Guide for the Care and Use of Agricultural Animals in Research and Teaching, 3rd ed’ (John McGlone, Janice Swanson, et al., 2010) [43]. We chose lactating Holstein cows with similar numbers of lactations (median = 3, range = 2 to 4) and days in milk (DIM) (median = 6 d, range = 3 to 10 d) between January and May 2016 from a 10,000-cow dairy farm located in Changchun City (Jilin Province, China).

The total incidence rate of LDA in the dairy farm was about 4%, and 210 cows were suspected LDA patients during the sampling period. Early postpartum cows with obvious clinical symptoms of LDA and no symptoms of other perinatal diseases were selected as the suspected LDA cows, and early postpartum cows without clinical symptoms of both LDA and other perinatal diseases were selected as the suspected healthy cows. Accordingly, 50 suspected LDA and 30 healthy early postpartum cows were preselected. The veterinarians conducted daily inspection of the cows, which were classified as suspected LDA according to the following symptoms [44]: (1) reduced appetite, disordered digestion, and mushy feces; (2) sunken right side of the waist and enlarged left side of the abdomen below the 11th rib arch; (3) a pinging sound through simultaneous auscultation and percussion on the abdomen area between the 9 and 13 ribs, marked by a line from the tuber coxae to the point of the elbow; and (4) rectal examination revealing medially displaced rumen and left kidney in diseased cows. All suspected LDA and healthy cows were subjected to routine physical examination to ensure absence of other co-morbidities. Cows were housed in a climate-controlled barn with individual tie stalls to reduce environmental interference. Cows had ad libitum access to the same diet offered twice a day (08:30 h and 16:00 h) and fresh water that was constantly supplied. Average DMI of each cow was calculated from data of consecutive 5 days before surgery. Blood samples of cows were extracted from the jugular vein in the morning (08:20 h) and centrifuged at 1,200 g for 15 min to obtain serum, which was stored at − 80 °C until analysis. Cows were milked twice daily at 08:00 h and 15:30 h and average milk yield of each cow was calculated from data of consecutive 5 days before surgery. The basic description of the cows is shown in Table 1.

After blood sample collection and DMI and milking data collection from preselected 50 suspected LDA and 30 suspected healthy early postpartum cows, surgery (right flank pyloric omentopexy, the paralumbar nerve conduction anesthesia combined with invasive anesthesia was adopted) was performed to confirm LDA cases in the suspected LDA group and 8 suspected LDA cases were excluded for lack of typical abomasum displacement; the incidence rate of ketosis high in local dairy farms in Changchun, 6 suspected LDA and 5 suspected healthy cows were excluded according to their serum BHBA concentrations (hematological diagnostic criteria of ketosis: serum BHBA > 2.0 mM). In addition, 6 suspected LDA and 5 healthy cows showed mild mastitis symptoms at the end of the data collection, thus these cows were also excluded from data analysis. Finally, data from 30 LDA and 20 healthy early postpartum Holstein cows were selected for data analysis. Health of all the cows after surgery in our experiment was monitored by recording rectal temperature, daily milk yield, and daily feed intake for at least 7 d until their recovery. The cows were finally released into the normal herd after experiment.

### Determination of blood biomarkers

Serum concentration of glucose (GLU), non-esterified fatty acids (NEFA), and β-hydroxybutyric acid (BHBA) were determined in a lab-based analysis using a Hitachi 7170 autoanalyzer (Hitachi, Tokyo, Japan) with commercial kits (BHBA: Cat No RB1008; NEFA: Cat. No. FA115; GLU: Cat No GL3815; Randox Laboratories, Crumlin, UK). Serum triglycerides (TG) and total cholesterol (TC) levels were measured using respective enzymatic kits (TG: Cat No E1003; TC: Cat No E1005-125; Applygen Technologies Inc, Beijing, China). Serum insulin (INS) level was measured with a bovine-specific insulin ELISA kit (Cat no: ENZ-KIT141-0001, Enzo Life Sciences, Inc, New York, USA) according to the manufacturer’s protocols. Serum glucagon (GC) level was measured with a Bovine Glucagon ELISA kit (Cat no: 2,040,013, Bioaim Scientific, Inc, Scarboroug, Canada) according to the manufacturer’s protocols. Activities of serum aspartate aminotransferase (AST), alanine aminotransferase (ALT), lactate dehydrogenase (LDH) and gamma-glutamyl transpeptidase (GGT) were determined with an automatic biochemical analyzer (Sekisui Medical Co., Ltd., Tokyo, Japan) using commercially available kits (AST and ALT: Cat no AS1204; LDH: Cat no LD3842; GGT: Cat no GT523; Randox Laboratories, Crumlin, UK) according to the manufacturer’s protocol. Levels of total protein (TP), albumin (ALB), globulin (GLO), blood urea nitrogen (BUN) and creatinine were measured using standardized kits supplied by Gcell (TP: Cat no GS0911G/B; ALB: Cat no GB0920G; BUN: Cat no GB9310S; creatinine: Cat no GB300S; Beijing Strong Biotechnologies, Inc., Beijing, China) by a Celercare® V2 automatic biochemical analyzer (MNCHIP Technologies Co., Ltd., Tianjin, China). Serum concentrations of total bilirubin (TBIL), direct bilirubin (DBIL), indirect bilirubin (IBIL) were detected by commercial ELISA Kits (TBIL: Cat no XK-SJH-1609; DBIL: Cat no XK-SJH-1608; IBIL: Cat no XK-SJH-1610; Shanghai Yuke Biological Technology Co., Ltd, Shanghai, China) according to the manufacturer’s protocols. The serum concentrations of Na, Cl, Ca, P, Mg, K, Cu, and Zn were determined by established procedures of a Beckman Synchron CX system (Beckman Instruments Inc., Fullerton, CA, USA). All the samples were assayed in duplicate.

### Statistical analysis

Results are expressed as the mean ± SEM. Data were analyzed using one-way ANOVA followed by Student’s test (SPSS 13.0 software; SPSS Inc., Chicago, IL). A *P*-value < 0.05 was considered statistically significant. ROC curves were constructed and each evaluated variable including area under the curve (AUC), sensitivity, specificity, and cut-off point was calculated to evaluate their suitability as warning sign for LDA. The AUC provides a useful metric to compare different biomarkers, with values close to 1 indicating an excellent predictive biomarker and a random line close to the diagonal (AUC = 0.5) having no diagnostic utility. An AUC value close to 1.00 is always accompanied by satisfactory values of specificity (SP) and sensitivity (SE) of the biomarker. The Youden Index (YI) was calculated as YI = Sensitivity- (1-specificity). A cut-point for each parameter was chosen when a maximum YI was detected. The revised quantitative insulin sensitivity check index (RQUICKI) was calculated as follow: RQUICKI = 1/ (log10 (glucose in mg/dL) + log10 (insulin in mU/mL) + log10 (NEFA in mmol/L)).

## Data Availability

The datasets used and/or analyzed during the current study are available from the corresponding author on reasonable request.

## Abbreviations

ALB: Albumin
ALT: Alanine aminotransferase
AST: Aspartate aminotransferase
BHBA: β-hydroxybutyric acid
BIL: Bilirubin
BUN: Blood urea nitrogen
DBIL: Direct bilirubin
GC: Glycagon
GGT: γ glutamyl transpeptidase
GLO: Globulin
GLU: Glucose
IBIL: Indirect bilirubin
INS: Insulin
LDA: Left displaced abomasum
LDH: Lactate dehydrogenase
NEB: Negative energy balance
NEFA: Non-esterified fatty acid
RQUICKI: Revised quantitative insulin sensitivity check index
TBIL: Total bilirubin
TC: Total cholesterol
TG: Triglyceride
TP: Total protein
